# Endovenous (minimally invasive) procedures for treatment of varicose veins: The gentle and effective alternative to high ligation and stripping operations

**DOI:** 10.1007/s00105-019-04532-y

**Published:** 2020-03-02

**Authors:** Karsten Hartmann

**Affiliations:** Venenzentrum Freiburg, Zähringer Str. 14, 79108 Freiburg, Germany

**Keywords:** High ligation and stripping, Radiofrequency RFA, Laser ablation EVLA, Mechanochemical ablation, Venous glue, Krossektomie und Stripping, Radiofrequenz RFA, Laserablation EVLA, Mechanochemische Ablation, Venenkleber

## Abstract

Thermal ablation of saphenous vein varicosis has developed into a standard procedure for treatment of varicose veins. The clinical success of the endovenous thermal procedure is comparable to high ligation and stripping operations and a significant difference between these groups could not be detected in long-term analyses. The only difference is in the genesis of saphenofemoral recurrence detected by duplex ultrasound: neoangiogenesis occurs after high ligation and stripping operation and after endovenous ablation of the great saphenous vein a recurrence occurs predominantly via a residual anterior accessory saphenous vein (AASV). Reduction of costs by an increase in endovenous procedures carried out in an outpatient setting in comparison to stripping operations, which are still frequently carried out in Germany (in comparison to other countries) as an inpatient procedure, have meanwhile been confirmed. An endovenous crossectomy (i.e., high ligation) should be strived for. Nonthermal endoluminal catheter procedures are predominantly reserved for treatment of the short saphenous vein.

## Abstract (de)

Die thermische Ablation der Stammvarikose hat sich zu einem Standardverfahren in der Behandlung des Krampfaderleidens entwickelt. Der klinische Erfolg der endovenös-thermischen Verfahren ist vergleichbar mit der Stripping-Operation, ein signifikanter Unterschied zwischen diesen Gruppen konnte in Langzeitanalysen nicht nachgewiesen werden. Lediglich in der Genese der mit Duplexultraschall-detektierten Krossenrezidive gibt es einen Unterschied: Eine Neoangiogenese tritt nach Stripping-Operation auf, ein Rezidiv über eine belassene V. saphena accessoria anterior vorwiegend nach endovenöser Ablation der Stammvene. Kosteneinsparungen durch vermehrt ambulant durchgeführte endovenöse Verfahren im Vergleich zur Stripping-Operation, die immer noch (im Vergleich zu anderen Ländern) in Deutschland oft stationär durchgeführt wird, sind inzwischen nachgewiesen. Eine endovenöse Krossektomie sollte angestrebt werden. Nichtthermische endoluminale Katheterverfahren sind vorwiegend der Behandlung der V. saphena parva vorbehalten.

Endoluminal procedures, in particular endovenous thermal vein ablation, are nowadays an established component in the treatment spectrum of varicose veins in Germany. In the Anglo-American world, thermal vein ablation is even given preference over stripping operations due to the high effectiveness, application safety, and high patient satisfaction [1]. The most widely used procedures are radiofrequency ablation (RFA) and laser ablation (EVLA). The RFA and EVLA procedures were both introduced in 1998/1999 and initially used with low wavelengths (EVLA) and low temperatures (RFA). The EVLA procedure was first used with bare fibers and a wavelength of 810–980 nm, and an improvement in effectiveness and reduction of side effects could be achieved with the introduction of radial fibers and lasers with higher wavelengths. The introduction of higher temperatures and segmental ablation also brought improvement in the effectiveness and tolerability of RFA.

The great advantage of endoluminal procedures in contrast to stripping operations is that they are minimally invasive. The next day, patients can undertake normal physical activities often including sport, and as a rule with no impairment due to the previous endovenous operation. Despite these undeniable advantages of endoluminal procedures, in recent times there has been increasingly more support for reinstating crossectomy and stripping (also known as high-ligation and stripping, HL+S) as the gold standard for treatment of varicose veins, because it leads to less saphenofemoral recurrences detected by duplex ultrasound (DUS) after 5 years in comparison to endothermal procedures [2]. This is particularly due to the fact that the currently available long-term data for endovenous thermal procedures are still taken from studies carried out during the initial stages of EVLA and RFA treatment [3]. These procedures worked in particular with too little energy or imprecise catheter positioning; however, long-term analyses on the clinical success (not saphenofemoral recurrence detected by DUS!) showed no significant differences between HL+S and thermal procedures [4, 5].

It is now known that a long residual saphenofemoral stump promotes recurrence [6, 7]. It is also known that in endovenous procedures, recurrence preferentially occurs via the anterior accessory saphenous vein (AASV) [4, 8, 9]. Therefore, the trend is towards an endovenous crossectomy, i.e., the treatment of the long saphenous vein is carried out up to the saphenofemoral junction, which again was not previously possible with bare fibers but which is no longer a problem with the modern radially radiating laser fibers and modern RFA techniques [10]. Whether prophylactic occlusion of the AASV in the region of the saphenofemoral junction should be promoted, as has always been the standard in surgical crossectomy, must be shown in further studies. An initial study on this topic is currently being prepared by the working group on endovenous treatment of the German Society for Phlebology.

## Endovenous treatment procedures

The endovenous procedures can be subdivided into two groups:

- thermal procedures that damage the veins by heat and occlude them, and
- nonthermal procedures that occlude the veins by a chemical process.

Thermal procedures include radiofrequency and laser procedures

**Fig. 1:**
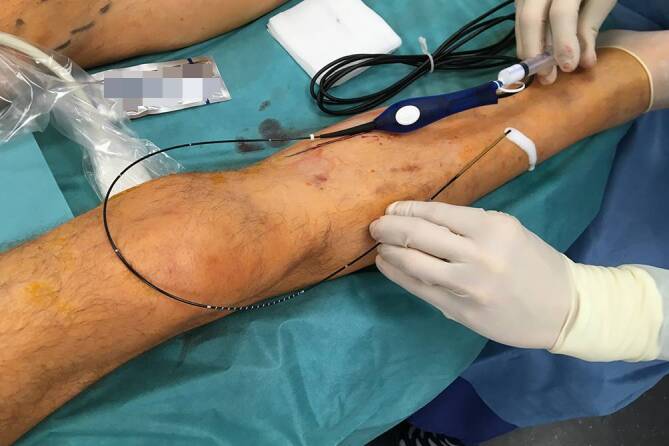
Foam sclerotherapy by a segmental radiofrequency ablation (RFA) catheter

Thermal procedures include radiofrequency and laser procedures. The most frequently applied radiofrequency procedures in Germany are segmental procedures, by which the veins are heated to 120 °C in sections, and radiofrequency-induced thermal treatment procedures, by which the veins are heated to 60–100 °C. In segmental RFA, sclerotherapy (e.g., a saphenofemoral recurrence) can additionally be undertaken via the lumen of the catheter ([11]; Fig. 1).

**Fig. 2:**
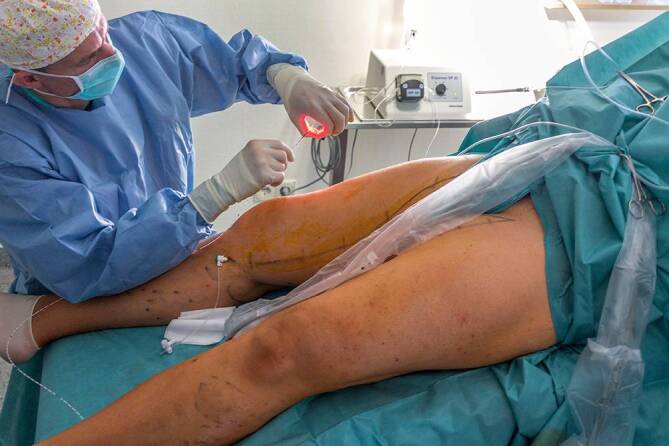
Endovenous laser ablation (EVLA) with a radially radiating probe

For laser procedures the tendency is towards wavelengths with a high absorption by water (1470–1940 nm). In this case radially radiating laser probes are preferred (Fig. 2), which lead to homogeneous damage of the vein wall. The temperature achieved by a 1470-nm laser with a radial probe is around 120–140 °C (±20 °C) [12, 13]. With theses radial probes, also a saphenofemoral recurrance with a persiting saphenofemoral stump can nowadays be accurately occluded. [10].

The nonthermal procedures include mechanochemical ablation (MOCA), the cyanoacrylate (glue) closure system, and in an extended sense, duplex-controlled foam sclerotherapy of the saphenous veins.

**Fig. 3:**
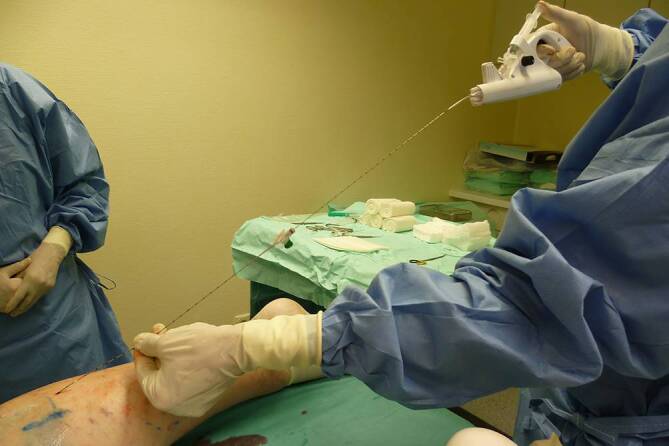
Mechanochemical ablation (MOCA)

The MOCA procedure is used to seal the vein by a combination of mechanical effects (catheter tip rotates and leads to slight painless damage to the intimal layer of the vein wall) and chemical action (an obliteration agent is injected into the vein below the catheter tip, Fig. 3).

**Fig. 4:**
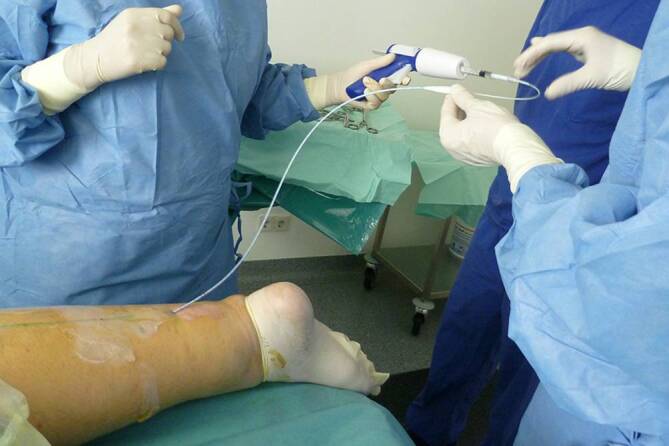
Vein adhesive procedure

In the glue closure system the vein is occluded with a special vein glue (monocyanoacrylate, Fig. 4).

The advantages of nonthermal procedures are particularly relevant in the treatment field of the short saphenous vein, because here there are many nerves lying close to the vein and no nerve damage can arise using nonthermal procedures. However, the occlusion rate is significantly lower than with thermal procedures (anatomical success 82% after 1 year and 80% after 2 years [14, 15]), except for with the cyanoacrylate (glue) closure system (occlusion rate after 2 years 95%, [16]), but it must be added that the adhesive is presumably not degraded (or only very slowly over many years) and remains in the vein as an “implant.”

## Endovenous crossectomy

**Fig. 5:**
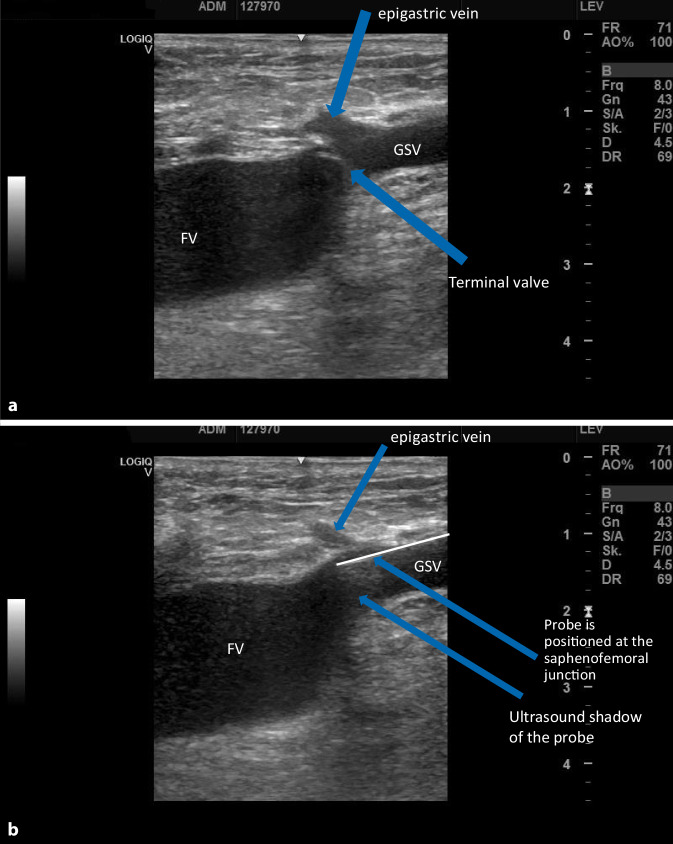
Duplex ultrasound: positioning the probe at the saphenofemoral junction. Endovenous crossectomy with evenly distributed occlusion of the long saphenous vein. **a** Before insertion of the probe. **b** Probe is in position. *FV* femoral vein, *GSV* great saphenous vein

**Fig. 6:**
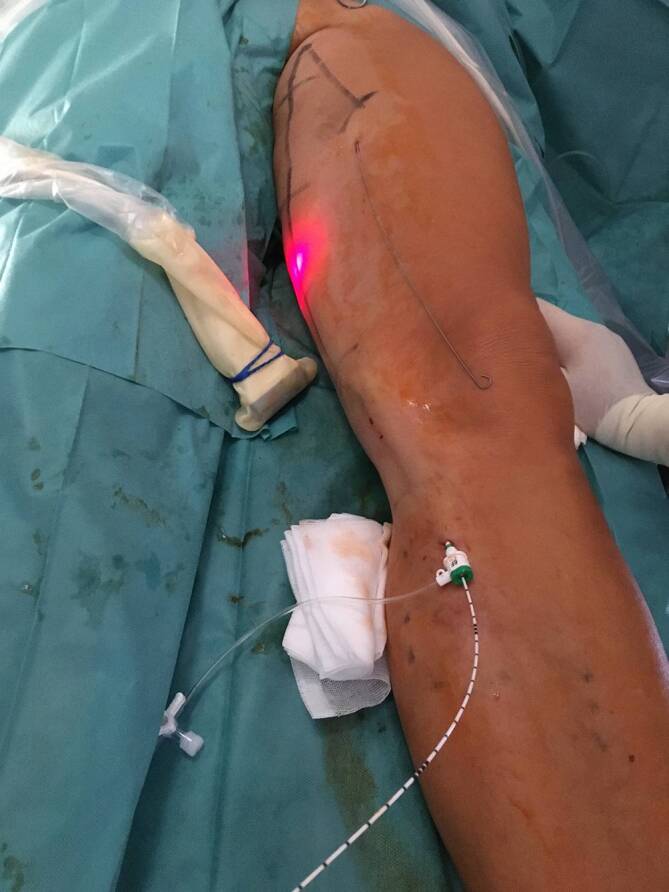
Treatment of the great saphenous vein (GSV; probe is inserted in the GSV) and simultaneous treatment of the anterior accessory saphenous vein (wire for cotreatment in the second stage is already inserted)

The probe is positioned at the saphenofemoral junction (Fig. 5a, b). This is particularly successful with radiofrequency devices and the new lasers with radially radiating probes. Prerequisites are a great deal of experience and very good knowledge of duplex ultrasound by the surgeon. The difference to surgical crossectomy is particularly that in endovenous crossectomy, the saphenofemoral junction with the epigastric vein remains patent (or reopens postoperatively). All other junctional branches which flow into the femoral vein via the long saphenous vein are immediately closed or are jointly closed via another puncture in a second step (anterior and, if necessary, posterior accessory saphenous veins, Fig. 6). Therefore, it is justified to speak of an endovenous crossectomy. The side branches flowing into the femoral vein directly in the region of the junction with the long saphenous vein remain patent; however, it is still unclear whether the recurrence rate is relevantly influenced by these smallest branches (larger branches can be simultaneously endovenously treated if necessary).

**Fig. 7:**
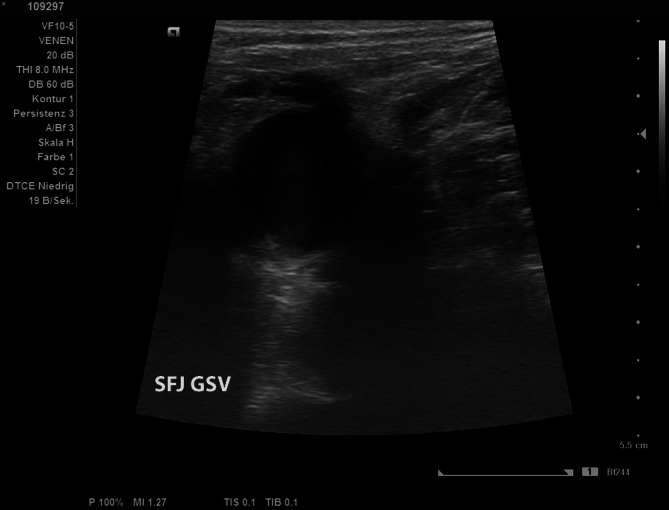
Duplex ultrasound: GSV junction 1 year postoperative with obliterated long saphenous vein without stump, the epigastric vein is open and sufficient. (*R* right). *SFJ* saphenofemoral junction, *GSV* great saphenous vein

The thermal treatment of the long saphenous vein up to the junction with the femoral vein results in occlusion of the complete long saphenous vein in the region of the saphenofemoral junction. The region up to the epigastric vein often becomes recanalized in the area of the saphenofemoral junction, but the terminal valve of the long saphenous vein remains closed or is sufficiently thermally damaged with shrinkage of the junctional funnel of the long saphenous vein that no recurrence can occur (Fig. 7).

## Complications of endovenous procedures

Complications following endovenous procedures are very rare but can also be severe. Frequent side effects are hematomas and ecchymoses, which occur in particular due to incorrect infiltration of the tumescent local anesthesia with perforation of the vein and when using bare laser fibers. Hyperpigmentation along the length of the treated vein is a rare occurrence and can regress within 1 year [17, 18]. Secondary bleeding and wound infections are rare as no large cutaneous incisions should be made (no crossectomy and also surgical venous access should be avoided whenever possible).

Deep vein thrombosis of the leg is very rare after treatment [19]; however, there is a danger of appositional thrombus growth in deep veins [20]. A post-ablation thrombus extension (PATE) has been observed [21] in the postoperative phase in approximately 1% of patients after endovenous techniques. In cases of PATE I a follow-up control is carried out within 1–2 weeks, with no medicinal treatment, but in cases of PATE II–III, therapeutic anticoagulation is initiated. As a rule, an apposition thrombus is degraded within 2–4 weeks and controls are carried out with duplex sonography until complete degradation [21]. The classification of PATE is given in Table 1.

**Table 1:** S2k guidelines on diagnostics and treatment of varicose veins of the German Society for Phlebology

PATE	Anatomical location	Procedure/treatment
0	Expansion of the thrombus up to the deep vein (=even occlusion = desired treatment success)	No specific measures necessary
I	Expansion of the thrombus a few millimeters into the deep vein with narrowing of the lumen of the deep vein up to 25%	Duplex ultrasound control (every 1–2 weeks) until regression of thrombus (level 0) Consideration of anticoagulation in prophylactic doses
II	Expansion into the deep vein with narrowing of the lumen up to a maximum of 50%	Duplex ultrasound control (every 1–2 weeks) until regression of thrombus (level 0) Therapeutic anticoagulation until regression of thrombus to level 0
III	Narrowing of the deep vein >50% without complete occlusion of the deep vein	
IV	Complete occlusion of the deep vein	Therapeutic anticoagulation analog to treatment of deep leg vein thrombosis Regular duplex ultrasound controls After thrombus regression (level 0) termination of therapeutic anticoagulation can be considered

*PATE* post-ablation thrombus extension

## Classification of PATE after endovenous treatment

Occasionally patients report phlebitic complaints in the treated area some days after an endovascular intervention. This occurs particularly in the treatment of large, very superficially lying varices. Further possible complications are:

- lymphedema (aggravated),
- matting,
- pathological scar tissue formation (keloids),
- injury of deeper-lying vessels during an intervention.

A complication of thermal procedures are nerve lesions. These are rare and often reversible [17, 18]. The danger from nerve lesions increases the more distal the endovenous treatment is carried out on the lower leg [22]. The possibility of skin burns must be mentioned; however, these only occur when skin protection with perivascular tumescent local anesthesia is insufficient.

A side effect of cyanoacrylate (glue) closure procedures is incomplete occlusion of the treated vein with recanalization in the peripheral region. In this case, complete occlusion can be achieved in a second step with duplex-controlled foam sclerotherapy; however, this is not always successful.

Extensive knowledge of the anatomy is helpful when performing endovenous procedures (as, for example, acquired from open varicose vein surgery) [23].

## Data situation and discussion

In a meta-analysis by Hamann et al. [4] from 2017, a total of 3004 studies on the topic of HL+S versus endoluminal procedures (EVLA, RFA, and foam sclerotomy) were included, of which 12 could be used in the meta-analysis. The definition of a successful operation in the groups was no reflux in the treated vein after 5 years (anatomical success) and showed no significant difference between HL+S in comparison to EVLA and RFA as well as no significant difference in the occurrence of saphenofemoral recurrence detected by DUS. A significant difference in effectiveness could only be shown in comparison to foam sclerotomy; however, the meta-analysis showed that when a saphenofemoral recurrence occurred, this differed between HL+S and EVLA/RFA. After HL+S, neoangiogenesis tended to occur in the saphenofemoral region, whereby after EVLA/RFA, a recurrence occurred most frequently via the AASV [4].

The clinical success rate is very high for both procedures, with no significant differences

The number of saphenofemoral recurrences detected by DUS is similar for surgical and endovenous procedures; however, in surgical crossectomy there is a new formation of varicose vessel in the junction (so-called neoangiogenesis) and in endovenous procedures a recurrence occurs via the AASV. The clinical success rate is very high for both procedures, with no significant differences.

A further study by Wallace et al. from 2018 [9] showed a 5-year anatomical success of 93% for EVLA and 85% for HL+S (*n* = 140 for each group). The number of saphenofemoral recurrences detected by DUS in this study did not differ between HL+S and EVLA, and the only difference was that in HL+S, neoangiogenesis occurred and in EVLA a recurrence via the AASV in the same proportions (around 15%) [9]. A meta-analysis by Kheirelseid et al. [5] also from 2018 showed no significant differences between HL+S in comparison to EVLA and RFA after 5 years.

The two large German studies on this topic, Flessenkämper et al. from 2016 [24] and Rass et al. from 2015 [25], are now considered. Both studies came to the result that in HL+S, significantly fewer saphenofemoral recurrences were revealed by DUS than in EVLA. For these studies it should be known that HL+S was carried out by outstanding surgeons from vein centers with longstanding experience. This is certainly not the general standard in Germany; however, in the early stages of the study, the laser technique was still in its infancy (data collection started in 2004/2005) and was carried out with low wavelengths (810 nm for Rass et al. and 980 nm for Flessenkämper et al.) and bare fibers were used. The laser energy applied in the study by Rass et al. was low and recanalization of the treated saphenous veins often occurred (62% of saphenofemoral recurrences detected by DUS!). In the study by Flessenkämper et al., in which the author of this article also participated (surgical and endovenous procedures), the laser energy was higher but the distance from the bare fibers to the junction was much more than 2 cm. The methodologically very well carried out studies of Flessenkämper et al. and Rass et al. therefore show that an HL+S carried out to a very high standard shows a low 5‑year risk of saphenofemoral recurrence of less than 10%; however, neither study showed a failure of endoluminal procedures but both clearly demonstrated that the laser power + energy as well as the distance of the laser fibers from the junction and the nature of the laser fibers play an important role for the result of endoluminal procedures.

In order to understand why not only the laser energy and power play a role in EVLA but also the wavelength and nature of the probe, the mechanism of action of EVLA must be scrutinized. There are five postulated mechanisms of action [26]:

1. direct contact between fiber tip and vein wall,
2. thermal interaction between laser light emitted out of the fiber tip and the vein wall:
  A. direct absorption by the vein wall of the light scattered by the blood that reaches the wall (increase in vein wall temperature),
  B. heating the blood surrounding the fiber tip by direct laser light absorption which causes heat flows to conduct towards the wall and, upon arrival, produce an increase in wall temperature
3. steam bubbles,
4. carbonization of the fiber tip,
5. coagulum formation of the blood.

In bare fibers there is more carbonization of the fiber tip and, therefore, the resulting temperature profiles are practically identical for different wavelengths [27]. Different modes of action are therefore not explainable by different wavelengths alone but more due to other parameters, such as the velocity of fiber retraction and power (watt). First with the use of radially radiating fiber tips and a defined retraction velocity could the carbonization of the fiber tip be mostly avoided and therefore the effect of the higher wavelengths with absorption by water could come into play. This means that the earlier effect of the hot needle, or the heating of the carbon on the fiber tip and therefore the damage of the vein wall up to perforation, is avoided and the laser light at 1470 nm, or now with a 1940 nm laser, heats the water in the vein wall (but of course also the erythrocytes) better. Therefore, a targeted, gentler, and even more effective occlusion of the treated vein is achieved. The use of linear endovenous energy density (LEED) alone for measurement of the energy released is in this case a senseless parameter: 50 J/cm can be achieved with 10 W and 2 mm/s retraction speed or with 0.1 W and 0.02 mm/s retraction speed (which would have no effect). Therefore, the additional information on power (watt), retraction speed, nature of the probe tip (e.g., radial or bare), and the wavelength used is essential.

The use of outpatient endovascular procedures reduces the total costs for treatment of varicose veins

The first 5‑year data with respect to the 1470 nm laser with a radial probe and segmental RFA were published by Lawson et al. in 2018 [8], with 97% (EVLA) or 96% (RFA) anatomical success. In 15% of the cases, a DUS-detected saphenofemoral recurrence occurred over the AASV after 5 years (*n* ~ 171 per group). This is a further indication of the importance of the AASV in treatment of the saphenofemoral junction.

A further interesting aspect of endovenous procedures is the consideration of the cost structure. The endovenous procedures are carried out to an increasing extent in an outpatient setting. Nevertheless, it is maintained that the endovenous procedures are more expensive in comparison to stripping operations, which are still often carried out under inpatient conditions [2]. The proportion of outpatient operations in Germany is still clearly lower than in countries such as the USA, Canada, and Scandinavian countries [28]. Compared to inpatient interventions, outpatient operations offer many advantages: savings in personnel and hospital costs, a more patient-oriented treatment (e.g., due to reduced mental stress especially in older patients), the possibility of more flexible schedule planning, and a lower risk of nosocomial infections [29–32]. In 2018, Jacob and Walker [33] investigated the German MICADO selective contract, which enables a case-related remuneration for outpatient operations replacing inpatient operations, with respect to profitability. The result was that endovenous thermal operations of the saphenous veins develop an effect for replacing inpatient treatment, despite an increase in operations induced by the supply, and lead to a reduction of costs from the perspective of the statutory health insurance [33]. Due to the reduction of expensive inpatient stripping operations and increased use of outpatient minimally invasive endovenous procedures, the total costs for treatment of varicose veins are therefore reduced.

## Practical conclusion

- Thermal ablation of saphenous veins in particular has developed into a standard procedure in the treatment of varicose veins.
- The clinical success of the endovenous thermal procedure is comparable to the stripping operation.
- There is a difference only in the genesis of the duplex ultrasound (DUS) detection of saphenofemoral recurrence:
  - A neoangiogenesis occurs after stripping operations.
  - A recurrence via a remaining anterior accessory saphenous vein (AASV) after endovenous ablation of the saphenous veins.
  - Due to the apparent simplicity when carrying out the endoluminal procedure there is a great danger that in the long term a loss of quality in the method can occur. Therefore, endovenous procedures should be in the hands of experienced phlebologists and/or physicians with extensive knowledge in phlebology, who regularly participate in further training in phlebology.
  - Cost savings by outpatient endovenous procedures in comparison to stripping operations have now been confirmed.
  - An endovenous crossectomy should be strived for.
